# Wearable devices in pregnancy health care

**DOI:** 10.1016/j.ebiom.2025.105974

**Published:** 2025-10-08

**Authors:** eBioMedicine

Pregnancy is a highly complex and dynamic process. Although WHO recommends routine antenatal care for a positive pregnancy experience, it can still be hard to achieve, especially for working women who might not have the time or paid leave to allow them to visit hospital regularly. Remote monitoring at home during pregnancy was introduced into clinical practices more than a decade ago. Early remote monitoring can be helpful for women with pregnancy complications, such as hypertension and diabetes, yet implementation is often completed by specialised equipment that might not be broadly available. Wearable devices, such as smart watches and bands equipped with biological sensors, are capable of measuring physiological parameters. Whether these devices can provide more systematic real-time monitoring of maternal or fetal status and assist prenatal care are emerging topics attracting attention from both the academic community and society at large.

Body temperature, sleep, and heart metrics and physical activity data are the most common modalities collected by wearable sensors, and it might be helpful to monitor these physiological changes over the course of the pregnancy. Any abnormal changes in these metrics could be useful in guiding clinical care of the patient.

In the April, 2025 issue of *eBioMedicine**,* Young-Lin and colleagues retrospectively analysed sleep metrics collected by wrist-worn Fitbit devices from more than 2000 pregnant women. Young-Lin and colleagues found that total sleep time reached its peak at gestational week 10, but decreased for the remainder of pregnancy, whereas deep and rapid eye movement sleep decreased significantly throughout the course of pregnancy. A similar sleep pattern was also observed in another study published in the September, 2025 issue of *eBioMedicine**,* in which the investigators analysed daily resting heart rate (RHR), number of daily steps, and total minutes of daily sleep collected by the participants’ own wearable sensors from more than 100 pregnant women. The investigators observed that RHR reached a local peak in the first trimester around gestational week 5, and then gradually decreased until gestational week 9. After that, RHR increased steadily until 8–9 weeks before delivery, then dropped until birth to a level below pre-pregnancy. The number of daily steps decreased during the whole pregnancy, reaching the minimum in the first week postpartum. In addition, this study showed that RHR curves were correlated positively with oestrone, progesterone, and cortisol, but negatively with human chorionic gonadotropin, whereas total minutes of daily sleep showed the opposite correlations. The researchers also found evidence that RHR patterns of pregnancies ending in stillbirth or miscarriage differed from those ending in livebirth. However, it is important to note that only nine pregnancies in the cohort did not end in a livebirth, and larger studies will be essential to understand this in greater depth. For other biometrics, a study published in *npj Digital Medicine* in August, 2024, based on 97 full term pregnancies, revealed that nightly peak temperatures increased compared with pre-pregnant temperature in the first trimester, and then slowly decreased across the rest of pregnancy, whereas nightly trough temperatures only increased in the third trimester until delivery. Nightly peak respiratory rate, on the other hand, remained stable throughout the pregnancy. Nightly peak heart rate variability decreased from conception until a few weeks before the day of delivery, a trend also reported by an earlier study published in the June, 2022 issue of *JMIR mHealth uHealth*. All these studies show the feasibility of wearable devices to provide a non-invasive method to monitor pregnancy-related physiological and behavioural changes, and might help health providers to understand the pregnancy process more comprehensively.

An emerging question is whether we can use wearable-derived data to assist the treatment of pregnancy complications. In a randomised controlled trial (RCT) published in the November, 2024 issue of *American Journal of Obstetrics & Gynecology*, the authors tested whether a periodic mobile application (eMOM) with wearable devices can improve maternal and neonatal outcomes among women with diet-controlled gestational diabetes without additional guidance from health-care personnel. The investigators found that, compared with patients receiving standard care, patients in the intervention group (standard care plus eMOM with a continuous glucose monitor, an activity tracker, and a food diary 1 week per month until delivery) showed a lower mean change in fasting plasma glucose and lower capillary fasting glucose levels. The intervention group reported more vegetable intake and engaged in more light physical activities than the control group. The risk of newborns with macrosomia was also lower in the intervention group. These results suggest that wearable devices might help to improve maternal and neonatal outcomes in clinical practices by supporting healthy dietary and physical activity behaviours.

The use of wearable devices, as with all new technology, also has the potential for negative effects. For example, there is the possibility that continuous health data monitoring might lead to anxiety, such as reported in a small study of cases in patients with chronic heart diseases in July, 2020 issue of *Journal of Medical Internet Research*. In addition, as most wearable devices available to consumers are not equipped with medical-grade technology, their long-term accuracy might not be sufficient for robust data analyses. Inaccurate measures could also potentially lead to maladaptive health behaviours, either missing the optimal treatment timeframe (false normal measures) or increasing the burden of health-care service (false abnormal measures). Taken together, the application of customer wearable sensors should be considered with the guidance of clinical practitioners during pregnancy.

The more details we know about pregnancy and its complications, the better placed we are to improve health outcomes. The use of wearable devices to monitor pregnancy is still early in development. With the integration of more sensitive and accurate sensors and more powerful AI-supported algorithms, this approach shows promise in supporting real-time monitoring of pregnancy and in assisting diagnosis and treatment of pregnancy complications. In addition, prospective RCTs are necessary to fully demonstrate efficiency in reducing pregnancy complications and improving outcomes. *eBioMedicine*, as a translational publishing platform, welcomes studies about the utilisation of wearable devices in pregnancy health care and other clinical practices.

